# Mitogenome of the blood feeding leech *Haementeria acuecueyetzin* (Hirudinida: Glossiphoniidae) from Tabasco, Mexico

**DOI:** 10.1080/23802359.2020.1814888

**Published:** 2020-09-08

**Authors:** V. M. Sosa-Jiménez, G. Torres-Carrera, A. Manzano-Marín, S. Kvist, A. Oceguera-Figueroa

**Affiliations:** a Posgrado en Ciencias Biológicas, Universidad Nacional Autónoma de México, Ciudad de México, Mexico; b Departamento de Zoologıa, Instituto de Biologıa, Universidad Nacional Autonoma de Mexico, Ciudad de México, Mexico; c Centre for Microbiology and Environmental Systems Science, University of Vienna, Vienna, Austria; d Department of Ecology and Evolutionary Biology, University of Toronto, Toronto, Canada; e Department of Natural History, Royal Ontario Museum, Toronto, Canada

**Keywords:** Blood feeding leech, Southern Mexico, Clitellata, Annelida

## Abstract

Here, we present the mitogenome of the blood feeding leech *Haementeria acuecueyetzin* (Hirudinida: Glossiphoniidae) based on specimens collected in Tabasco, Mexico. The circular genome is 14,985 bp in length, and consists of 13 protein-coding genes, 22 tRNA genes, two rRNA genes, and an AT-rich control region. Phylogenetic analysis based on the 13 protein-coding genes and two rRNA genes places *H. acuecueyetzin* sister to *H. officinalis* within the family Glossiphoniidae. Mitochondrial gene order in *H. acuecueyetzin* is consistent with other members of Clitellata with no evidence of gene gain/loss, duplication, or rearrangement.

*Haementeria acuecueyetzin* Oceguera-Figueroa (2008) is a blood-feeding leech that inhabits large parts of southern Mexico. It feeds on a variety of vertebrates including cattle, manatees, crocodiles, and even humans (Oceguera-Figueroa 2008; Pérez-Flores et al. 2016; Charruau et al. 2020). The genus includes 13 species geographically restricted to the Neotropics (Oceguera-Figueroa 2012; Oceguera-Figueroa and León-Règagnon 2014). *Haementeria* species have been used as model organisms in studies of neurophysiology (De-Miguel et al. 2001), development (Sawyer et al. 1981), and bacterial symbioses (Manzano-Marín et al. 2015), among other areas.

On 18 March 2018, a total of 30 specimens of *H. acuecueyetzin* were collected in Tabasco, Mexico (17° 40′ 56.53″N, 93° 00′07.92″W). For a parallel project regarding the leech bacterial endosymbionts, total DNA of a mixed pool of 120 bacteria bearing organs or bacteriomes from 30 leeches (four from each individual) was extracted using a commercial extraction kit (DNeasy Blood & Tissue Kit, Qiagen, Hilden, Germany). DNA libraries were constructed using the NGS Nextera FLEX DNA library preparation kit (Illumina Inc., San Diego, CA) according to the manufacturer’s protocol and total DNA was multiplexed together with 11 other samples and sequenced on a single lane on the HiSeqX platform (150 bp paired-end) at SickKids, Toronto, Canada. Adapters were trimmed with the FastX-Toolkit (Gordon and Hannon 2010), and sequencing quality was assessed with FastQC v0.11.9 (Andrews 2019). Reads were assembled using SPAdes v3.13.0 (Bankevich et al. 2012) and, subsequently, taxonomic assignment of each contig was performed by a local BLASTx search (Altschul et al. 1997) against the proteomes of *Providencia siddallii* (CVRF01000001–CVRF01000004), *Helobdella robusta* (AF178680) and *Haementeria officinalis* (LT159848) as reference for bacterial endosymbiont, host (leech), and leech mitochondrial sequences, respectively. We recovered a linear contig of ∼15 Kbp with strong similarity in both nucleotide identity and length when compared to *Haementeria officinalis*, the only previously sequenced mitogenome from a congener. Online BLASTn and BLASTx searches confirmed the leech affinity of the contig and its components (https://blast.ncbi.nlm.nih.gov/Blast.cgi). Shorter sequencing reads were mapped to the single contig using Bowtie 2.3.5.1 (Langmead and Salzberg 2012) and reassembled, resulting in a sequence of >15 Kbp in length and a coverage of 1085x. Low complexity regions at both 5′- and 3′-ends were trimmed for a final sequence of 14,985 bp in length. The resulting sequence was annotated using the MITOS web server (Bernt et al. 2013) with one round of manual curation to correctly define 5′- and 3′-ends of genes, resulting in a complete mitogenome consisting of 13 protein-coding genes, 22 tRNA genes, two rRNA genes, and an AT-rich control region. The newly reported mitogenome was deposited in GenBank under accession number MT683771. Voucher specimens for *H. acuecueyetzin* are deposited in the Colección Nacional de Helmintos, Universidad Nacional Autónoma de México, Mexico City, Mexico (CNHE 11386).

Sequences of the 13 protein coding genes and two ribosomal RNAs were used to infer the phylogenetic position of *H. acuecueyetzin* within Hirudinea using all available mitogenomes from GenBank. A maximum-likelihood tree was obtained using RaxML v.8.2.4 (Stamatakis 2006), employing the model GTRGAMMAI and setting bootstrap replicates as autoMRE function. Phylogenetic analysis places *H. acuecueyetzin* as the sister taxon to *H. officinalis*, within a clade exclusively formed by other members of Glossiphoniidae. The glossiphoniid clade, in turn, is the sister to the rest of hirudineans (Figure 1). Mitochondrial gene order in *H. acuecueyetzin* is consistent with that of other members of Clitellata with no evidence of gene gain/loss, duplication, or rearrangement.

**Figure 1.**
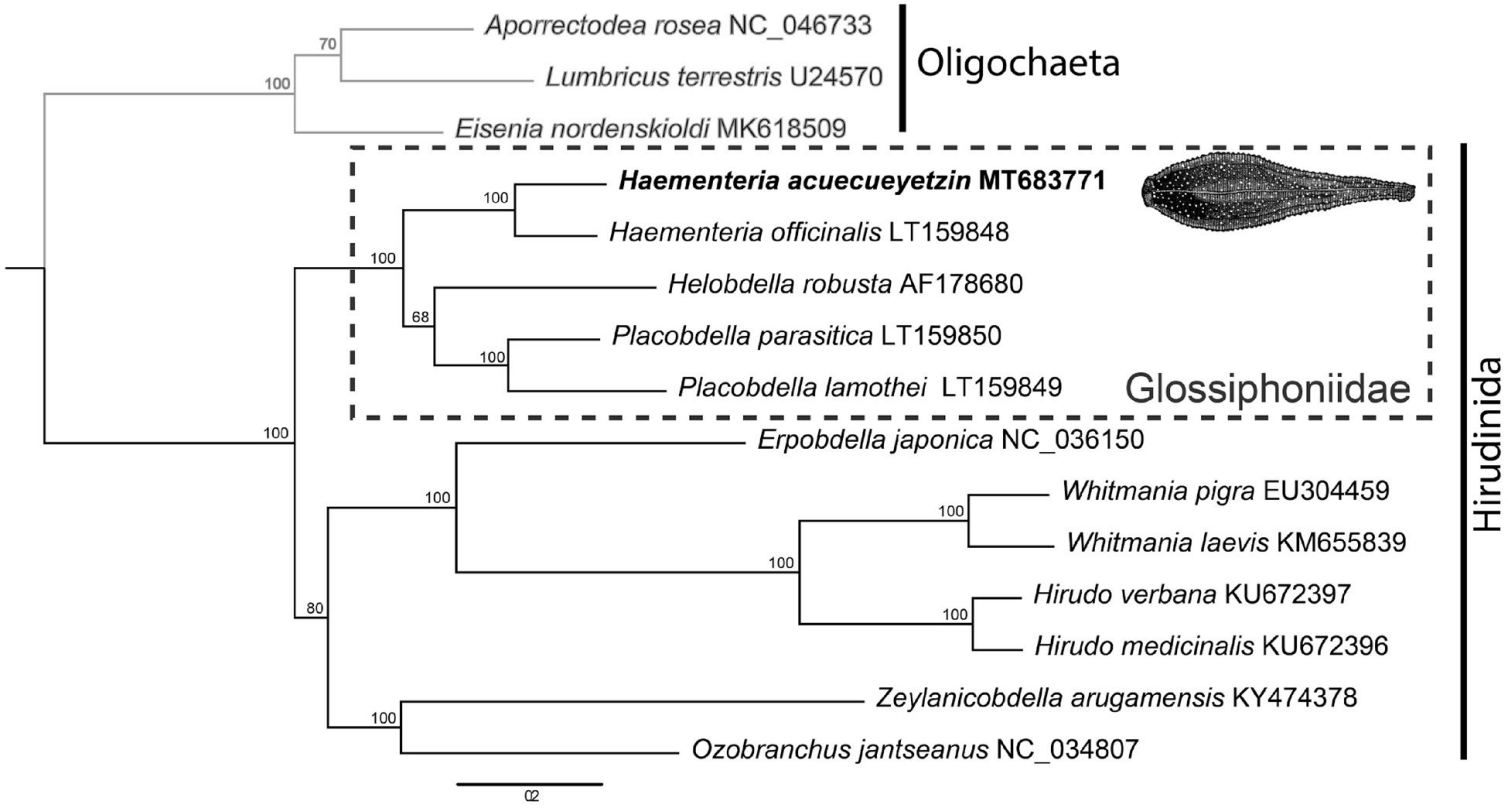
Phylogenetic hypothesis inferred by maximum-likelihood using 13 protein-coding genes and two ribosomal genes sourced from all available leech mitogenomes on GenBank. *Haementeria acuecueyetzin* places as the sister taxon to *H. officinalis* within a larger clade formed by members of Glossiphoniidae (dotted box). The ingroup (Hirudinida) is represented by black branches and Oligochaeta (outgroup) in gray branches.

## Data Availability

The data that support the findings of this study are openly available in GenBank of NCBI at https://www.ncbi.nlm.nih.gov/genbank/, reference number MT683771.
